# Particulate Matter Exposure in a Police Station Located near a Highway

**DOI:** 10.3390/ijerph121114541

**Published:** 2015-11-13

**Authors:** Yu-Cheng Chen, Chin-Kai Hsu, Chia C. Wang, Perng-Jy Tsai, Chun-Yuan Wang, Mei-Ru Chen, Ming-Yeng Lin

**Affiliations:** 1 National Environmental Health Research Center, National Health Research Institutes, 35 Keyan Road, Zhunan Town, Miaoli 350, Taiwan; E-Mail: yucheng@nhri.org.tw; 2 Department of Industrial and Information Management, College of Management, National Cheng Kung University, Tainan 701, Taiwan; E-Mail: jimmyg83525@yahoo.com.tw; 3 Department of Chemistry, College of Science, National Sun Yat-sen University, Kaohsiung 804, Taiwan; E-Mail: chiawang@mail.nsysu.edu.tw; 4 Department of Environmental and Occupational Health, College of Medicine, National Cheng Kung University, Tainan 701, Taiwan; E-Mail: pjtsai@mail.ncku.edu.tw; 5 Department of Administration Police, College of Justice Administration, Central Police University, Taoyuan 333, Taiwan; E-Mail: g885422@seed.net.tw; 6 Department of Occupational Safety and Health, College of Medicine and Life Science, Chung Hwa University of Medical Technology, Tainan 717, Taiwan

**Keywords:** particulate matter, ultrafine particles, police station, highway

## Abstract

People living or working near roadways have experienced an increase in cardiovascular or respiratory diseases due to vehicle emissions. Very few studies have focused on the PM exposure of highway police officers, particularly for the number concentration and size distribution of ultrafine particles (UFP). This study evaluated exposure concentrations of particulate matter (PM) in the Sinying police station near a highway located in Tainan, Taiwan, under different traffic volumes, traffic types, and shift times. We focused on periods when the wind blew from the highway toward the police station and when the wind speed was greater than or equal to 0.5 m/s. PM2.5, UFP, and PM-PAHs concentrations in the police station and an upwind reference station were measured. Results indicate that PM2.5, UFP, and PM-PAHs concentrations in the police station can be on average 1.13, 2.17, and 5.81 times more than the upwind reference station concentrations, respectively. The highest exposure level for PM2.5 and UFP was observed during the 12:00 PM–4:00 PM shift while the highest PAHs concentration was found in the 4:00 AM–8:00 AM shift. Thus, special attention needs to be given to protect police officers from exposure to high PM concentration.

## 1. Introduction

Particulate matter exposure can result in adverse health effects such as cardiopulmonary diseases [1,2]. Among them, PM2.5 (aerodynamic diameter ≤2.5 μm), ultrafine particles (UFP, aerodynamic diameter ≤100 nm), and polycyclic aromatic hydrocarbons (PAHs) have recently drawn significant attention due to their increasingly known adverse effects in in human health. Elevated PM2.5 concentration has been linked to increased mortality and shortened lifespan [3,4], while UFPs, due to their small size and large number concentrations, can penetrate deep into the lungs, resulting in pulmonary inflation and even lung destruction [5,6,7]. Furthermore, UFP can enter the blood and lymph circulation, reaching sensitive targets such as bone marrow and the heart [8]. PAHs exposure can result in DNA damage [9,10] and even lead to lung cancer [11]. High molecular weight PAHs (PM-PAHs, PAHs with three rings or greater, which are predominately adsorbed on particles) are primarily in the PM2.5 category, with many having been identified as mutagens and carcinogens [12,13].

Highway vehicle emissions are a major source of PM, especially UFP and PAHs [14,15,16,17,18]. Several studies have pointed out that highway maintenance workers or people living near highways are exposed to high levels of PM and noise, and as a consequence, are more vulnerable to cardiovascular diseases [19,20,21]. Other studies have found that highway toll booth workers are exposed to high concentration of PM2.5, UFP, and PAHs [22,23,24]. However, relatively few studies have focused on highway police officers, who spend most of their time working near highways. Most previous studies on the PM exposure of police officers have focused on traffic police officers, as summarized in Table 1. One study from India indicated a decrease in lung function among traffic police officers [25]. Another study from Beirut, Lebanon, pointed out that traffic police are more likely to develop cancer than office-working police due to daily traffic exposure [26]. In China, studies have found high PAHs levels among traffic police officers and a higher incidence in lifetime cancer risk [27,28] investigated the dispersion of UFP from the roadway to the surrounding workplaces; however, their study did not measure workplace UFP concentration during different working shifts, especially the size distribution. Size distribution and number concentration are important parameters for epidemiological studies and regulatory purposes; however, even fewer studies have focused on highway police officers’ PM exposure under different traffic volumes and time periods in police stations.

Currently, there are around 2000 highway police officers working near highways in Taiwan. As such, their working environments make them vulnerable to highway traffic PM pollution. Highway police officers typically work long hours (up to 10 h daily), which increases their exposure duration to PM. Moreover, many highway police officers also spend their off hours resting inside the police station, thereby further increasing their exposure to highway PM. In this study, we investigated the PM exposure in a police station immediately adjacent to the Sinying highway toll booth. At the toll booth, only three out of seven northbound and two out of seven southbound lanes used Electronic Toll Collection (ETC), while the rest of the lanes still used manual collection of highway tickets. In the manual collection lanes, passing traffic must stop completely to hand over highway passes to the highway toll collectors, then accelerate back to normal driving speed (60–110 km/h). These changes in driving speed could increase PM emissions, especially in the UFP range [29,30]. It should be noted that vehicles passing through the ETC lanes only have to slow down to 50km/h instead of stopping completely. To investigate how highway traffic affects the PM concentration in the police station, special attention was focused on PM2.5, UFP, and PM-PAHs during periods when the wind was ±45° perpendicular to the highway and the wind speed was ≥0.5 m/s.

**Table 1:** Overview of past pollutant exposure studies among traffic police officers.

City	Pollutant	References
Jalgaon, India	PM_resp_, NOx, SOx	[25]
Beirut, Lebanon	VOCs	[26]
Tianjin, China	PAHs	[27]
Grenoble, France	PM_resp_, PAHs, and aldehydes	[31]
Bangkok, Thailand	PM2.5 and PM10	[32]
Beijing, China	Particle and gas phase PAHs	[33]
Kathmandu, Nepal	PM10	[34]
Jakarta, Indonesia	PM2.5, PM10, UFP, CO	[35]
Milan, Italy	PM_resp_, CO, Benzene, Toluene, Ethyl-benzene, M-P Xylene, and O Xylene	[36]

## 2. Experimental Section

### 2.1. Description of Sampling Site

**Figure 1:**
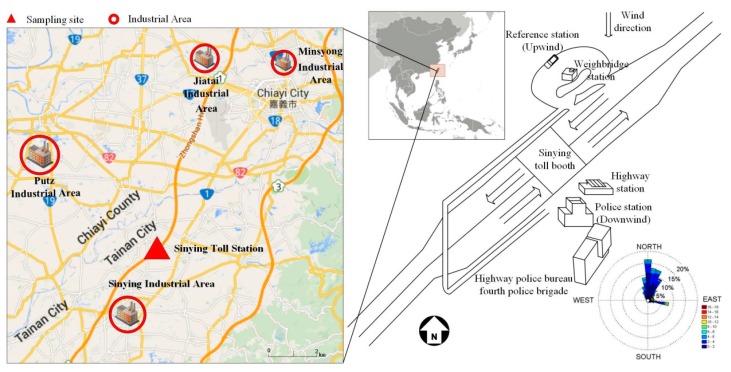
The left panel shows the surrounding areas of the sampling site at the Sinying police station, Tainan, Taiwan and four nearby industrial areas (Putz, and Sinying, Minsyong, and Jiatai in red circles). The right panel indicates the location of the two sampling sites, namely the police and reference stations in Sinying. A wind rose is also included in the figure.

To address PM air quality at the police station near the highway toll booth when the wind carries the highway traffic emissions, we selected the fourth police brigade Sinying Branch of the Highway Police Bureau (police station) (23.357948 latitude, 120.339121 longitude) and an upwind reference station for comparison in Tainan, Taiwan. Here, the police station and reference station are located approximately 50 m (south) and 30 m (north) from Highway No. 1, respectively (Figure 1). In addition to highway traffic emissions, there are other PM emission sources from the surrounding industrial areas (such as Putz, Sinying, Minsyong, and Jiatai) which might also contribute to the measured concentrations (Figure 1).

In the Sinying police station, there are 25 police officers working, consisting of one director, four sergeants, and 20 police officers who take turns tending the front desk, which is a four hour shift. Normally, each police officer works for a total of 10 h a day (comprising two to three shifts, with each shift being 2–6 h, and with rest time in between shifts), five days a week. It should be noted that the front door of the police station was left open during the entire sampling period. Thus, the building is naturally ventilated and the penetration factor would be close to one. Furthermore, only one row of bushes approximately 90 cm tall was between the highway and the police station. The Annual Average Daily Traffic (AADT) volume was around 72,000 vehicles based on monitoring at a Sinying toll station, consisting of around 30% and 70% of diesel and gasoline vehicles, respectively. (Taiwan Ministry of Transportation and Construction, 2013).

#### 2.1.1. Police Station

**Figure 2:**
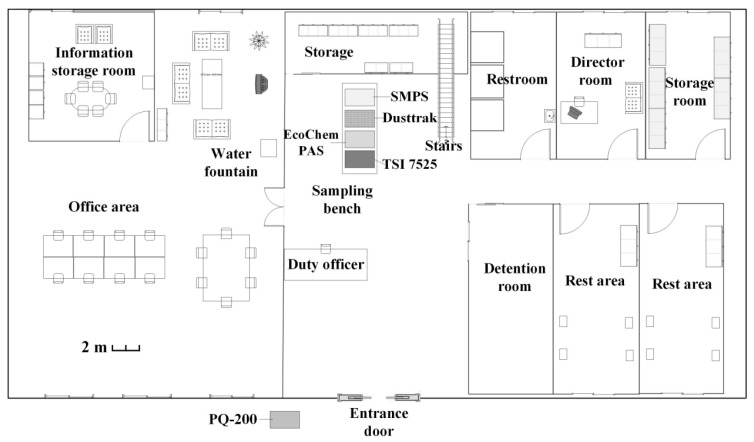
Layout of the police station. The instruments were placed on the sampling bench located right behind the duty officer and in front of door.

We simultaneously investigated the PM concentrations at the Sinying police station (downwind) and reference station (upwind) of the highway from 27 December 2013 1:00 AM to 1 January 2014 12:00 AM (Figure 1). Table 2 lists the instruments and time resolutions of the sampling used at the police station and reference station. For the police station, the inlet of the real-time instruments was made of 1/2ʺ stainless steel and was set 1.5 m above the ground to simulate the workers breathing height. We used a Scanning Mobility Particle Sizer (SMPS, DMA Model 3081 and CPC Model 3787, TSI Inc., Shoreview, MN, USA) to characterize the size distribution and number concentration of the particles ranging from 8 to 224 nm. A layout of the police station floor plan can be seen in Figure 2. The SMPS upscan, downscan, and retrace times were set to 90 s, 30 s, and 30 s, respectively, while the aerosol inlet flow rate was set to 1.5 L/min to avoid diffusional loss between the inlet and sensor. Real-time PM2.5 mass concentration was measured using DustTrak (model 8520, TSI Inc.) with a flow rate of 1.7 L/min. PM-PAHs concentrations were obtained using a PAHs monitor (model PAS2000 CE, EcoChem Analytics, League, TX, USA) with a flow rate set to 2 L/min. We also measured CO_2_ concentration using an indoor air quality meter (IAQ-Calc, model 7525, TSI Inc.). In addition, we placed an ambient fine particulate sampler (model PQ200, Mesa Labs, Inc., Butler, NJ, USA) 5 m away from the entrance door of the police station. The PQ200 had a flow rate of 16.67 L/min and was used to monitor the outdoor PM2.5 mass concentration. PQ200 samplers are often used for ambient PM2.5 sampling and are in accordance with the US EPA 40CRF Part53. Finally, a video recorder placed at the entrance of the police station was used to record the activities within the police station.

**Table 2:** Air monitoring instruments and locations.

Data	Instrument	Sampling Interval	Police Station	Reference Station	
PM (8–224 nm) physical properties (#/cm^3^)	SMPS	150 s	indoor	outdoor	
PM2.5 (μg/m^3^)	PQ200	12 h ^a^	outdoor	outdoor	
PM2.5 (μg/m^3^)	DustTrak8520	60 s	indoor	outdoor	
CO/CO_2_ (ppm)	IAQ-Calc	5 s	indoor	outdoor	
PM-PAHs (ng/m^3^)	PAS 2000	60 s	indoor	outdoor	
Meteorological data	Watch Dog 2550	60 s	NA	outdoor	

^a^ Filter-based measurement; Sampling Interval: time resolution of sampling; NA: not applicable.

#### 2.1.2. Reference Station

The instruments positioned in the reference station were similar to the ones in the police station. We deployed a van (Hyundai Grand Starex) as the reference station (23.360140 latitude, 120.339510 longitude), which was located in the parking area of the highway weigh station, as shown in Figure 1. The 1/2ʺ stainless steel sampling inlet protruded from the van’s rear window and was approximately 2 m above the ground. In this sampling platform, an SMPS (DMA Model 3081 and CPC Model 3010, TSI Inc.) with an inlet flow rate of 1 L/min was used for particle size distribution (8–224 nm), DustTrak (model 8520, TSI Inc.), PAHs monitor (model PAS2000, EcoChem), and IAQ-Calc (IAQ-Calc, model 7525, TSI Inc.) were also employed to measure PM concentrations. Meteorological data (wind speed, wind direction, temperature, dew point, RH and rainfall) were recorded using a weather station (model Watch Dog 2550, Spectrum Technologies Inc., Aurora IL, USA). A PQ200 for PM2.5 mass concentration was also stationed at the reference station.

### 2.2. Instrument Inter-Comparison and Quality Assurance

Every other day, we drove to the reference station van to the police station for a side by side inter-comparison of the two sets of instruments. These comparisons continued at least one hour. Upon obtaining the inter-comparison average ratio of the SMPS, we used Savitzky-Golay smoothing [37] to fit the average ratio. Then, we multiplied the reference station SMPS values by this average ratio to better compare the two SMPSs located at the two different places. For the other instruments (PM2.5, PM-PAHs, CO), the average ratios were only a constant value and did not require smoothing. We also synchronized the time of the two sets of instruments during the inter-comparison. In addition, we used a HEPA filter (Pall cooperation, New York, NY, USA) to check for leaks before and after moving the instruments for inter-comparison. It should be noted that the reference station SMPS broke down on 31 December 1:00 AM, while the police station PAS 2000 also did not function from 29 December 12:00 PM–31 December 12:00 AM. Lastly, the weather station broke down on 29 December from 2:37 AM–10:52 PM; therefore, we did not use the data during this period due to unknown wind parameters.

### 2.3. Data Analysis

PM exposure within the police station under different traffic volumes, types, and working shifts was investigated in this study. Real-time instruments were averaged to 5 min and 4 h for traffic and working shift analysis, respectively. Time dependent figures were used to investigate peaks and diurnal patterns of traffic and pollutants. We further investigated the exposure of the policemen working at the front desk, which follows a 4-h shift system (*i.e.*, 12:00 AM–4:00 AM, 4:00 AM–8:00 AM, 8:00 AM–12:00 PM, 12:00 PM–4:00 PM, 4:00 PM–8:00 PM, and 8:00 PM–12:00 AM). Pearson’s correlation coefficient was used to evaluate the association between variables.

## 3. Results

In this section, we first present the overall PM exposure (PM2.5, UFP, and PM-PAHs) at the police station and reference station; then, we discuss the PM exposure under different working shifts. Results of the particle size distribution and number concentration from the SMPS are also presented for different working shifts. Finally, we discuss how different traffic flow patterns affect the PM concentration within the police station.

### 3.1. Police Station and Reference Station Overall PM Exposure

Table 3 shows the descriptive statistics for the PM2.5, UFP, and PM-PAHs concentrations, as well as CO_2_, wind speed and traffic volumes during the measurement period for the police station and reference station. Here, PM2.5 mass, UFP number, and PM-PAHs concentrations obtained from the police station can on average be 1.13, 2.17, and 5.81 times higher than the upwind reference station, respectively. The mean PM2.5 concentrations from the DustTrak measurements at the police station and reference station were generally around 182 μg/m^3^ and 161 μg/m^3^, respectively. A high UFP number concentration (up to 9.18 × 10^4^ #/cm^3^) from the SMPS measurements with an average value of 1.90 × 10^4^ #/cm^3^ was observed in the police station, while the average concentration at the reference station was around 8.75 × 10^3^ #/cm^3^. The PM-PAHs concentration at the police station and reference station showed averages of around 55 ng/m^3^ and 9 ng/m^3^, respectively. It should be noted that the PAS-2000 sampler may respond differently to individual species, resulting in un-proportionality of sampler signals to the concentration of individual species [38].

**Table 3:** Statistical descriptions of PM2.5, UFP, PM-PAHs CO_2_, WS and Traffic volume obtained from the police station (PS), reference station (RS), and toll booth.

	PM2.5 (μg·m^−3^)		UFP dN/dlogdp (#/cm^3^)		PM-PAHs (ng·m^−3^)		CO_2_ (ppm)		WS (m·s^−1^)	Traffic (Vehicle/5 min)	
	RS	PS	RS	PS	RS	PS	RS	PS	RS	Toll Booth	
Minimum	35	60	2.28 × 10^3^	2.94 × 10^3^	0.0	1.10	462	465.7	0	26	
1st Quartile	112	138	5.73 × 10^3^	1.27 × 10^4^	2.0	29.5	490	503.2	0.56	108	
Median	136	167	8.38 × 10^3^	1.79 × 10^4^	5.0	47.5	500	514.9	2.22	251	
Mean	161	182	8.75 × 10^3^	1.90 × 10^4^	9.43	54.8	510	515.6	3.09	232	
3rd Quartile	176	210	1.05 × 10^4^	2.35 × 10^4^	12.0	73.1	526	526.6	4.45	325	
Maximum	447	444	2.64 × 10^4^	9.18 × 10^4^	427.0	255.4	636	689.0	16.7	517	
Standard deviation	79	77	3.90 × 10^3^	8.94 × 10^3^	15.0	34.3	23	17	3.15	121	

The CO_2_ concentration in the police station was slightly higher than the reference station, which could be attributed to emission sources and indoor air pollution. However, the maximum CO_2_ concentration of 689 ppm was still lower than Taiwan’s indoor air quality standards (1000 ppm for an 8-h average). The average wind speed was around 3 m/s, indicating that the wind was often light, not stagnant (<0.5 m/s), during the measurement period.

### 3.2. GLM Statistical Analysis of PM Exposure Level in the Police Station

Here we use repeated measures generalized linear model (GLM) to find the parameters that best describes the PM exposure level in the police station. We focus on the whole measurement period and during conditions when the wind is from the highway and the wind speed is larger or equal to 0.5 m/s.

#### 3.2.1. PM Exposure Level during the Whole Measurement Period

We first examine the parameters affecting the PM2.5, UFP, and PM-PAHs concentration in the police station during the whole measurement period. Meteorological data (*WS*, *WD*, *T*, and *RH*), traffic data (car and truck volumes), and time of the day (*i.e.*, 12:00 AM–4:00 AM, 4:00 AM–8:00 AM, 8:00 AM–12:00 PM, 12:00 PM–4:00 PM, 4:00 PM–8:00 PM, and 8:00 PM–12:00 AM, which has been categorized to coincide with working shift) were used as parameter inputs. Wind direction (*WD*) was derived into 12 classes, each with 30 degrees of amplitude: North (N), North-Northeast (NNE), East-Northeaster (ENE), East (E), East-Southeast (ESE), South-Southeast (SSE), South (S), South-Southwest (SSW), West-Southwest (WSW), West, West-Northwest (WNW), and North-Northwest (NNW). PM2.5 was significantly affected (*p* < 0.0001, *R*^2^ = 0.67) by *WS*, *WD*, time of day, and car volumes. UFP was significantly influenced by *WS*, *WD*, time of day, and truck volumes “(*p* < 0.001, *R*^2^ = 0.34). PM-PAHs was significantly affected by *WD*, time of day, and truck volumes (*p* < 0.0001, *R*^2^ = 0.46).

#### 3.2.2. PM Exposure Level during Downwind Conditions

During downwind conditions and when the wind speed is larger or equal to 0.5 m/s, we examine how meteorological data (*WS*, *T*, and *RH*), traffic data (car and truck volumes), and time of the day (grouped to coincide with working shift) affects the PM2.5, UFP, and PM-PAHs concentration in the police station. PM2.5 was significantly affected (*p* < 0.0001, *R*^2^ = 0.21) by *WS*, time of day, car volumes, *T*, and *RH*. UFP was significantly influenced (*p* < 0.0001, *R*^2^ = 0.45) by *WS*, time of day, and truck volumes. PM-PAHs was only significantly affected by time of day (*p* < 0.0001, *R*^2^ = 0.34). We will further discuss the effects of time of day (categorized to coincide with working shift) and traffic on PM2.5, UFP, and PM-PAHs in the following section.

### 3.3. Police Station PM Exposure on Working Shifts

**Figure 3:**
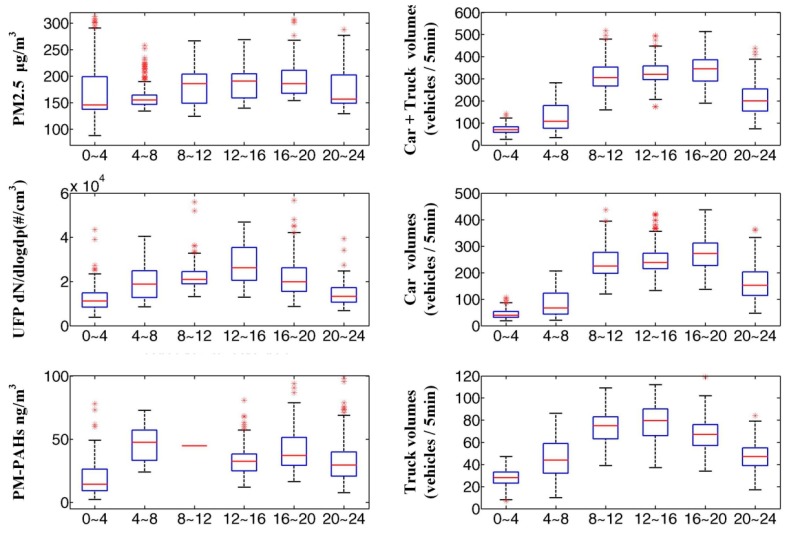
Diurnal pattern of the traffic volume, PM2.5, UFP, and PM-PAHs in the police station during different working shifts. The box represents the 25th and 75th percentiles while the whiskers represent the 5th and 95th percentiles.

Figure 3 shows the diurnal pattern of traffic volume, PM2.5, UFP, and PM-PAHs. Notice that the traffic volume for cars has two peaks (8:00 AM–12:00 PM and 4:00 PM–8:00 PM) while the truck volume has only one peak (12:00 PM–4:00 PM). The diurnal pattern of PM2.5 concentrations at the police station indicates that a higher PM2.5 concentration occurs during the daytime (8:00 AM–8:00 PM) as compared to nighttime (8:00 PM–8:00 AM) due to higher daytime traffic volumes. Higher daytime PM2.5 concentrations were also found in other indoor workplaces and near-roadway environments [39,40]. The UFP concentrations in the police station during the daytime was higher than the nighttime concentrations (Figure 3), which is also attributable to higher traffic volumes during the daytime. For the PM-PAHs diurnal pattern, two peaks occurred throughout the day, one at 04:00–08:00 and another at 16:00–20:00. This was attributed to the morning and afternoon traffic rush hours. It should be noted that data from 08:00 to 12:00 was insufficient for drawing the box and whiskers in the boxplot. Further discussion on the contribution of PM from traffic is presented in Section 3.4.

**Figure 4:**
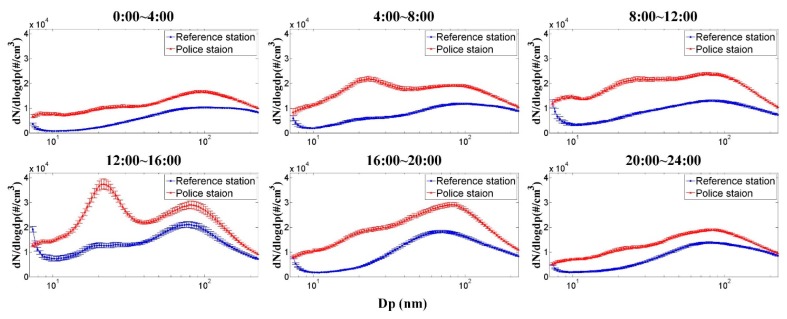
Average size distribution and number concentration from SMPS data for different four-hour working shifts at the police station and reference station. The error bar represents one standard deviation.

Figure 4 indicates the average particle size distribution and number concentration (8–224 nm) during different working shifts obtained from the police station and reference station. As can be seen, there are peaks at around 20–30 nm at the police station, but not at the reference station when the wind blew from the highway, especially during the daytime hours. This peak may originate from vehicle tailpipe emissions, which usually have a number median diameter of around 20 nm [41,42]. Furthermore, the peak is more obvious during the daytime as compared to nighttime due to the higher daytime traffic volume.

### 3.4. Inter-Correlation of Police Station PM Concentration

**Figure 5:**
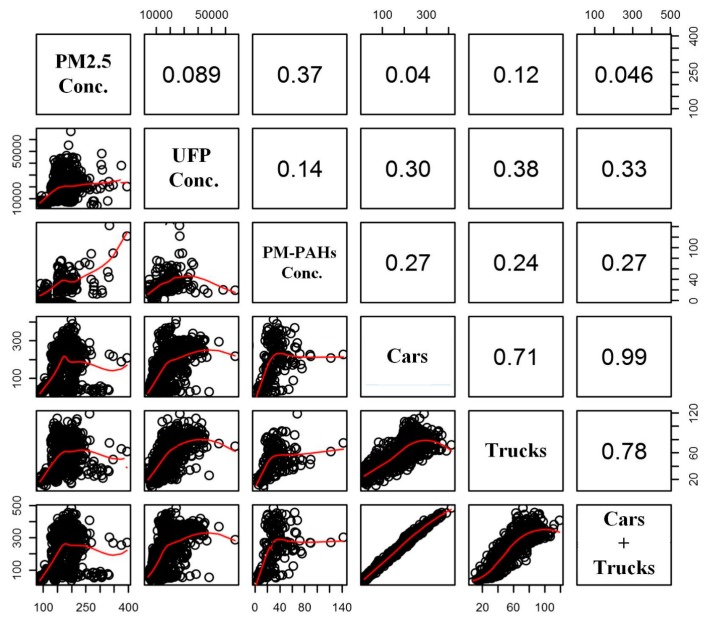
Inter-correlation between the vehicle data and pollution measured in the police station. The upper right corner shows the coefficient of determination and the red lines in the lower left corner represent loess curves.

The scatter plot of the traffic volume with PM2.5, UFP, and PM-PAHs concentrations for the police station is shown in Figure 5. In general, UFP measurements of the police station had high correlations (*R*^2^) using loess curve fit for car plus truck, car, and truck traffic volumes, followed by PM-PAHs and PM2.5. The higher *R*^2^ observed for the UFP and PM-PAHs measurements are attributed to direct vehicle emissions. Vehicle emitted UFP are characterized by large number concentrations, but small mass concentrations. Therefore, poor correlations between PM2.5 and traffic volume in the police station were expected. Studies have also indicated that UFP contribution to total PM mass concentration in near-roadway environments is minor [43]. Moreover, trucks with diesel engines could result in higher correlations with UFP than cars and overall traffic in this study. Previous studies have also demonstrated that the trucks with diesel engines emit more UFP, while most cars with gasoline engines release less PM2.5, UFP, and PM-PAHs [44,45].

**Figure 6:**
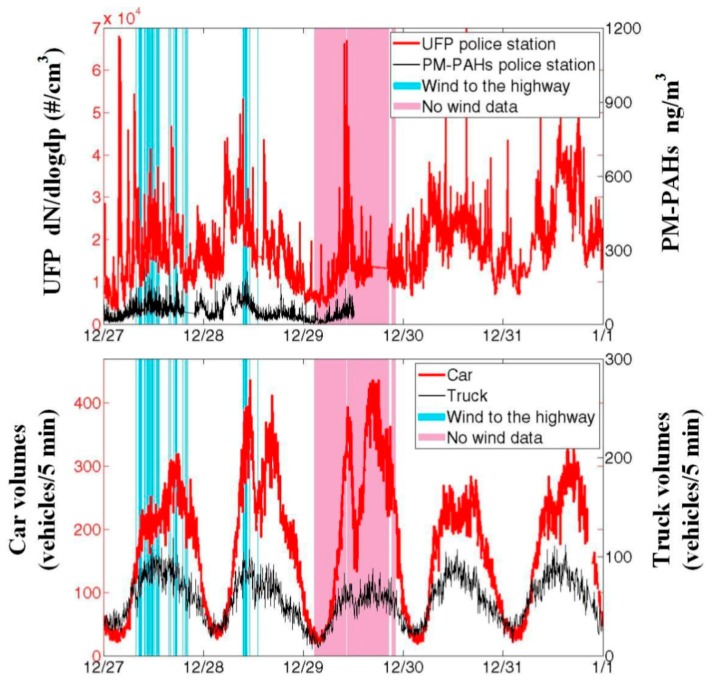
Time series plot of the UFP and vehicle volume throughout the study period. Periods of the color bars (light blue and pink) were not used for analysis since they indicate the wind direction was not from the highway or the data was missing.

Figure 6 shows the time series plot of the traffic volume and UFP for police station measurements during the sampling period. As shown, UFP concentrations increase during traffic rush hours when the wind blows from the highway. This is also true for PM-PAHs, for which the trend is similar with traffic volume. Thus, the source of the UFP and PM-PAHs is confirmed to mainly originate from the highway vehicle emissions. In addition, the number of incidental peaks was greater in the police station as compared to the reference station, which could be attributed to smoking near the police station entrance or gas stoves used for boiling water in the police station.

## 4. Discussion

### 4.1. PM Exposure Comparison with Previous Research

PM2.5 concentrations from DustTrak are much higher than the Taiwan indoor and outdoor air quality standard, which states that the PM2.5 24-h averages should both be below 35 μg/m^3^. However, it should be noted that previous studies have shown that the DustTrak can overestimate the PM mass concentration by a factor of 3 [46,47,48]. The PQ200, set in front of the police station and at the reference station, registered an average PM2.5 concentration of around 61 μg/m^3^ and 48 μg/m^3^, respectively. Therefore, we may be overestimating the PM2.5 exposure at the police station from the DustTrak measurements.

These measured UFP concentrations were in accordance with other studies with near-roadway measurements, for which high UFP concentrations (10^4^~10^5^ #/cm^3^) were also reported [49,50]. Moreover, UFP diurnal trends were similar to previous studies from Beijing, China [51] and Helsinki, Finland [52].

### 4.2. Health Risk Implications

Police workers as well as people who are living busy roadways are exposed to high concentration of air pollution such as PM2.5, UFP, and PM-PAHs, especially people who are living in the downwind section. If possible, future urban planning should consider the location layout of busy roadways and residential areas to lower the public’s exposure to hazardous air pollutants. The public should also be informed of methods that can be taken to protect them from busy roadway air pollution. Methods include understanding and avoiding the air pollutant peak hours, applying ventilation control, and using respiratory protection devices.

## 5. Conclusions

Applying real-time instruments can provide “qualitative” exposure concentrations of particles for highway police officers during various working shifts. Police officers working at police stations near busy highways are exposed to high PM concentrations, not only during their duty shifts, but also while spending time resting in the police station. High PM2.5 concentrations consistently over 100 μg/m^3^ were observed in this study using the DustTrak at both the police station and reference station, while PM2.5 levels from PQ-200 at the police station (61 μg/m^3^) and reference station (48 μg/m^3^) were above daily air quality standards (35 μg/m^3^). Moreover, high UFP concentrations up to 9.18 × 10^4^ #/cm^3^ were observed in the police station. Traffic volume was found to best correlate with UFP, followed by PM-PAHs and PM2.5 in the police station. As of the time of this study, many workplace UFP exposure limits are not well regulated globally, and only specific UFP species such as carbon nanotubes and titanium oxide are regulated by EU legislation and US Occupational Safety and Health Administration (OSHA). Mean PM-PAHs concentrations were 54.8 ng/m^3^ in the police station, while PM-PAHs concentrations, on average, were 9.43 ng/m^3^ at the reference station. The highest PM2.5 and UFP concentrations were observed during the 12:00 PM–4:00 PM shift. Given the high PM2.5, UFP, and PM-PAHs concentrations in the police station, more attention should be devoted to lowering the PM exposure. Respiratory protection devices are recommended at peak traffic hours, especially when the wind blows from the highway. Further toxicological and epidemiological studies are also needed to clarify the effect of PM on workers employed near busy roadways. One major limitation of this study is the use of the DustTrak and PAS 2000 instruments: although they can capture real-time pollution concentrations, the measured values require further verification with federal reference methods. Finally, while the construction work of the ETC was still ongoing during the time of the study, the effect of ETC on reducing vehicle emissions requires further study.
